# Digital Narratives: The Impact of Instagram^®^ on Mothers of Children with Congenital Toxoplasmosis

**DOI:** 10.3390/children11101267

**Published:** 2024-10-19

**Authors:** Gabrielle Gonçalves Veloso, Giovanna Cristina Machado-Kayzuka, Rhyquelle Rhibna Neris, Ana Carolina Andrade Biaggi Leite, Nayara Gonçalves Barbosa, Heloísa Cristina Figueiredo Frizzo, Gabrielle Vedoveto Escaliante, Adriana Moraes Leite, Beatriz Volpin Gomes Beato, Poliane da Silva Santos, Flávia Gomes-Sponholz, Lucila Castanheira Nascimento

**Affiliations:** 1 Ribeirão Preto College of Nursing, University of São Paulo, Ribeirão Preto 14040-902, Brazil; gabriellegve@gmail.com (G.G.V.); giovanna.machado@usp.br (G.C.M.-K.); rhyquelle@usp.br (R.R.N.); gabyve@usp.br (G.V.E.); drileite@eerp.usp.br (A.M.L.); beatrizbeato@usp.br (B.V.G.B.); polianesilva2001@usp.br (P.d.S.S.); flagomes@usp.br (F.G.-S.); 2 Department of Health Sciences, Public University of Navarre, 31006 Pamplona, Spain; anacarolina.andrade@unavarra.es; 3 Department of Maternal, Child and Psychiatric Nursing, School of Nursing, University of São Paulo, São Paulo 05403-000, Brazil; nbarbosa@usp.br; 4 Occupational Therapy Department, Federal University of Triângulo Mineiro, Uberaba 38025-180, Brazil; heloisa.frizzo@uftm.edu.br

**Keywords:** maternal and child health, toxoplasmosis, congenital, child, exceptional, online social networking

## Abstract

Background: Congenital toxoplasmosis leads to severe complications in childhood and presents significant global health challenges. In Brazil, the prevalence of toxoplasmosis during pregnancy and congenital cases ranges from 0.3 to 8 per 1000 live births. The clinical manifestations of congenital toxoplasmosis can include neurological and ocular damage, among other health issues, which place significant burdens on affected families. Objective: This study aims to investigate the experiences and motivations of mothers of children with congenital toxoplasmosis who share their journeys on social media, particularly Instagram. Methods: A qualitative virtual ethnography was used that explored the content shared by mothers of children diagnosed with congenital toxoplasmosis, aiming to understand how social media aids in their coping strategies and the support networks they create. Results: Fifteen Instagram accounts were analyzed, and twelve mothers participated in in-depth interviews. Thematic analysis revealed three main themes: the initial search for information and support, the evolving role of social media in advocacy and education, and the creation of a supportive online community. Conclusions: This study highlights the potential of social media to provide emotional support, disseminate information, and foster communities among mothers facing similar challenges, ultimately contributing to better care strategies and professional awareness for supporting families dealing with congenital toxoplasmosis.

## 1. Introduction

Congenital infections represent a global public health problem due to their high morbidity and mortality. They have the potential to impair fetal development, compromising the functioning of different organs and systems [1], as well as causing neurological sequelae, developmental delays, and other complications in childhood [2]. Congenital toxoplasmosis results from vertical transmission of the infection acquired during pregnancy [3,4]. The timing of maternal infection significantly affects the risk and severity of congenital infection. If a woman contracts toxoplasmosis early in pregnancy, particularly in the first trimester, the risk of transmission to the fetus is relatively low (around 10–15%) [5,6]. However, if transmission occurs, the fetal effects are often severe, including spontaneous abortion, stillbirth, or severe neurological and ocular damage. On the other hand, if the infection occurs later in pregnancy, particularly near delivery, the risk of transmission increases dramatically (up to 60–90%). Still, the consequences for the fetus are typically milder, often resulting in subclinical infection or less severe manifestations, such as chorioretinitis or subtle neurological abnormalities that may appear months or years after birth [5,6].

In some states of Brazil, the seroprevalence of toxoplasmosis during pregnancy ranges from 42 to 91%, with a risk of acquiring primoinfection during pregnancy of 4 to 6% [7]. Transmission of toxoplasmosis occurs mainly through ingesting raw or undercooked meat containing viable cysts, consuming water, fruit, vegetables, and seafood, and contact with soil contaminated by oocysts from cat feces, as well as through blood transfusion [3,8]. The high prevalence of toxoplasmosis during pregnancy in Brazil is influenced by a combination of environmental factors and varying levels of health education for both the population and the healthcare team. While some educational campaigns are in place, increased and more targeted public health initiatives are essential to effectively mitigate this significant health risk [8].

Prenatally, maternal infection can be confirmed through serological testing, specifically measuring IgG, IgM, and IgA antibodies against *Toxoplasma gondii*. IgG avidity testing can also be used to assess the timing of infection, as low avidity suggests a recent infection, while high avidity indicates an older infection. Amniocentesis, followed by PCR of the amniotic fluid, is the gold standard for detecting fetal infection, with higher sensitivity and specificity when performed after 18 weeks of gestation. Postnatal diagnosis in the neonate can involve serological testing to differentiate maternal from congenital infection by observing persistent IgG and specific IgM or IgA antibodies. Additionally, neonatal PCR testing, imaging (ultrasound and MRI), and ophthalmologic evaluation may be employed to detect early signs of congenital toxoplasmosis [9].

Although congenital toxoplasmosis is a preventable condition, its prevalence in Brazil ranges from 0.3 to 8 cases per 1000 live births [7]. Clinical manifestations include hydrocephalus, macrocephaly or microcephaly, intracranial calcifications, intellectual disability, sensorineural deafness, chorioretinitis, cataracts, and nystagmus, among other ocular alterations, epilepsy, hepatosplenomegaly, prematurity, and intrauterine growth restriction [3,8]. Around 35% of affected children have neurological clinical manifestations, including hydrocephalus, microcephaly, and intellectual disability, 80% have eye damage, and around 40% have hearing loss [8].

Families expecting a healthy newborn often face emotional and psychological challenges when confronted with the reality of a child affected by congenital infection. This discrepancy between expectation and reality can significantly impact the family dynamic, leading to stress and adjustment difficulties [10,11]. This situation can have an impact on the family system [11], especially regarding the care of these children, who may have limited physical or mental functions, dependence on medication, a specialized diet, and technology, and the need for rehabilitation therapy and multidisciplinary care [10], characterizing these children as those in the group of Children with Special Health Needs (CRIANES). These children require more attention and monitoring from health services than other children in the same age group [12].

Experiences in caring for children with congenital infections involve solving concrete problems and daily dilemmas about affective aspects, reconfiguration of life projects [10,13], the economic and financial impact, the search for care in different services, uncertainties regarding the future and development of the child, feelings of fear and sadness, as well as social prejudice regarding the child’s condition [10,13,14]. In caring for their child, many mothers experience distancing from their social, cultural, and leisure activities, in addition to the fragility of the support network and care-sharing [10,14].

In stressful situations, social media can be used to search for information and obtain social and emotional support, helping to mitigate difficulties [15]. Social media groups can offer opportunities for interaction between mothers, providing an alternative resource for support [15]. The internet can provide a sense of intimacy through anonymity, which helps people to share everyday experiences. The strength of virtual grouping is that it can intervene in the direction of research on a particular disease and public policies [16]. From this perspective, this study aims to analyze the motivations of mothers of children with congenital toxoplasmosis for creating and sharing their daily experiences on social networks and the meaning they attribute to creating these profiles for the care of these children. It is believed that delving deeper into this subject could help to implement caring strategies for mothers and families who care for children with congenital toxoplasmosis, as well as contribute to the qualification and awareness of professionals who provide support to families.

## 2. Materials and Methods

### 2.1. Study Design and Setting

This qualitative study employed virtual ethnography as a methodological framework and reflexive thematic analysis. Virtual ethnography, an anthropological research method, extends beyond traditional cultural activities, behaviors, and interests [17]. This approach aims to explore and understand the meanings behind various forms of communication and expression mediated by technology on digital media platforms [18]. By enabling inter-subjective engagement with the study subjects, virtual ethnography examines the relationships and interactions within these digital environments, providing deeper insights into people’s experiences and social processes [19,20].

The setting for the study was cyberspace, in which we sought to systematically observe Instagram profiles related to congenital toxoplasmosis to analyze content related to mothers caring for children with the pathology. Moreover, to ensure the study’s rigor, comprehensiveness, and credibility, we used the COREQ (Consolidated Criteria for Reporting Qualitative Research) as a guide to reporting the essential elements [21].

### 2.2. Recruiting Participants

The participants were selected through an electronic search on Instagram^®^, using the keywords congenital toxoplasmosis, toxoplasmosis in pregnancy, and toxoplasmosis. The snowball technique was also used. We included accounts in Portuguese from women over the age of 18 who are mothers of children diagnosed with congenital toxoplasmosis. We excluded accounts that only raised money for the child’s care without mentioning the routine or experiences of the diagnosis. The study’s objective primarily drove the decision to limit the search to accounts in Portuguese to focus on a specific linguistic and cultural context. This restriction allowed for a more homogenous sample in terms of language, which is crucial for maintaining consistency in the data collection and analysis process. Through the search, we found mothers of children with congenital toxoplasmosis who share and exchange information through this channel. Fifteen Portuguese-language accounts of mothers of children with congenital toxoplasmosis were included. This was the number of available accounts at the time of data collection, providing a clear understanding of the phenomenon and determining the conclusion of the data collection process.

To gain a deeper understanding of the meanings attributed to the publications, those responsible for the accounts identified were also invited to participate in a stage of interviews, which were carried out remotely. To take part in the interviews, the potential participants were contacted by one of the researchers via a private message on the social network. The message contained a personal introduction to the primary researcher and the study’s objectives. The women who indicated an interest in the study were given an appointment. Before the meeting began, the participants filled out the informed consent form (ICF) and an online sociodemographic form developed by the authors. Of the 15 accounts identified, 12 agreed to participate in this stage.

### 2.3. Data Collection

Data collection occurred between October 2022 and March 2023, with the researchers entering and immersing themselves in the virtual environment. The immersion in the virtual environment occurred by actively participating in social media, where the target population (mothers of children with congenital toxoplasmosis) shared their experiences. This involved regularly engaging with the content, reading discussions, and observing interactions, gaining a deeper understanding of the participants’ perspectives and experiences. This immersion was essential to ensure an authentic and nuanced interpretation of the data [18,20]. The author, G.G.V., a final-year undergraduate nursing student, read all the posts on the accounts included. Before extracting data, the author contacted the account holders, informing them of the study and asking for permission to collect from their posts and profiles. They also received a sociodemographic form to allow us a sample characterization. After permission, the data on the publications and characterization of the Instagram accounts were extracted from a form drawn up by the authors based on an instrument adapted from Frizzo [16]. This instrument included the code, title, date of creation, number of publications, description, year of publication on the topic, number of followers, number of accounts they are following, highlights, topics covered, and forms of expression, whether through photos, videos, reels, or stories. The data extracted at this stage were checked by the researchers G.M.K., L.C.N. and N.G.B., nurses with a doctorate or master’s degree and experience in qualitative research and congenital infections/family nursing.

The interviews, in turn, were conducted through individual online meetings between the participants and two researchers (G.G.V. and N.G.B.). Informed consent was obtained again from all participants before their involvement in the interviews. The interviews were recorded using the Google Meet platform. Unstructured interviews were conducted only once with each participant, lasting between 45 and 100 min, based on a script with open questions developed by the authors. The questions were related to daily experiences and dilemmas in caring for a child with congenital toxoplasmosis, repercussions on family dynamics, and experience with social networks. They were derived from a thorough review of the existing literature and a preliminary analysis of critical issues pertinent to the research topic. The iterative process of question development involved pilot testing during the first interview to refine and enhance the relevance and clarity of the questions.

### 2.4. Data Analysis

The data obtained from the publications and interviews were analyzed using the reflexive thematic analysis framework proposed by Braun and Clarke [22]. A researcher (G.G.V.) transcribed the interviews in full, and the research group subsequently reviewed the transcriptions. We adopted an inductive analysis approach, guided by the data rather than by a pre-established model or themes at a latent level. This approach aimed to go beyond the semantic content of the data by examining participants’ ideas, assumptions, and idealizations. The analysis followed six phases: (1) familiarizing with the data, (2) generating the initial codes, (3) searching for themes, (4) reviewing the themes, (5) defining and naming the themes, and (6) preparing the report (Braun and Clarke, 2021 [22]). The analysis was conducted by two researchers (G.G.V. and G.M.K.), with a view to methodological rigor and reducing the conceptual biases of those responsible (Braun and Clarke, 2021 [22]). The codes obtained in the first phase were checked by the senior researchers (L.C.N. and N.B.G.) and, after validation, they were organized into themes through research group meetings. Any disagreements between the authors were resolved at the meetings until a consensus was reached. Descriptive statistics provided detailed demographic information about our sample, offering context for the qualitative analysis of participants’ experiences with social media.

To maintain the anonymity of the participants, we used the code I (Instagram^®^), followed by the account number and the letter P (participant), according to the order in which the interviews were conducted.

### 2.5. Ethical Considerations

The study was conducted in accordance with the Declaration of Helsinki, and the protocol was approved by the university’s Research Ethics Committee under CAAE No. 58844322.9.0000.5393 on 15 June 2022. All participants signed the informed consent form when they agreed to participate in the study. To extract data from public accounts, the data were directly collected without the need to fill in the ICF, as per Resolution 510/2016. However, to maintain the privacy of the authors of the publications, names and other personal information were withheld.

## 3. Results

### 3.1. Characterization of the Participants

Fifteen Instagram^®^ accounts were analyzed, 14 of which were public and 1 private, with posts on the subject starting in 2014, with the number of posts varying between 17 and 5140. Of the 15 mothers responsible for the accounts analyzed, 12 agreed to participate in the interview phase. The participants were aged between 18 and 42 (μ = 32.8 years; σ = 6 years). Nine lived with partners or were married, while four reported non-traditional (or structurally diverse) compositions or did not answer the question. As for educational level, five had completed higher education, an essential factor for assessing the applicability of the results to other contexts. Concerning religion, 11 reported following some religious practices. Eight participants had access to filtered water, and eleven had a sewage system in their homes. Four of their children were premature, and all had sequelae related to congenital toxoplasmosis. The women did not specifically address the risk factors for infection during interviews, showing not to be sure how they became infected; however, their posts on social media frequently shared information on prevention and risk factors, with contamination from food and water being the most commonly mentioned concerns.

### 3.2. Thematic Analysis

**Figure 1:**
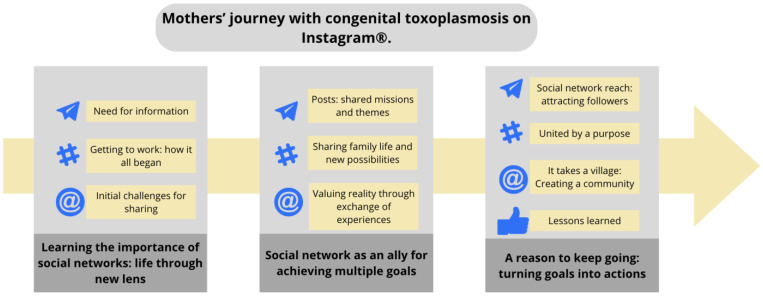
Analytical and descriptive themes representing the mothers’ journeys with congenital toxoplasmosis on Instagram^®^.

Based on the analysis of the codes found in the interviews and posts, three analytical themes were constructed, made up of their respective descriptive themes, represented in Figure 1 below.

#### 3.2.1. Analytical Theme 1: Understanding the Importance of Social Networks—Life under a New Lens

The news of the diagnosis of toxoplasmosis comes as a shock, awakening the need to search for information related to mothers, and social networks are potential spaces for changing courses and realities.

(a) Need for information at the time of diagnosis

The diagnosis of toxoplasmosis was often accompanied by surprise and a lack of knowledge about the characteristics and consequences of the disease. This knowledge gap led mothers to search for answers and information online. The images and results found were imprecise and not very generalizable, making it difficult for mothers to identify this information with their real-life situations. In this context, social networks emerged as a way of finding more reliable accounts. The search, however, was not easy, and the scarcity of accounts related to toxoplasmosis once again highlighted the knowledge gap of this audience: *“I confess to you that about toxoplasmosis, there is very little [content]. You see myelomeningocele, you see about cerebral palsy, Down’s Syndrome, but there’s very little about toxoplasmosis.”* (P1)

(b) Hands-on: how it all began

Amid the whirlwind of emotions experienced with the diagnosis, social networks were created to overcome the moment faced. They were seen as a means of strengthening, as they could express feelings of anger, self-forgiveness, and overcoming, positively shaping their battle. Some mothers chose to continue using their own social media accounts to talk about toxoplasmosis. In contrast, others saw the need to start new toxoplasmosis-themed profiles and focus more on the diagnosis, sharing the child’s day-to-day life. In these moments, many mothers used their posts to give a voice to their own children’s experiences, writing from their point of view: *“Thank you to everyone who is following me, who is interested in my story. This was Mom’s way of letting off steam and telling me how I’m the miracle in her and Dad’s lives.”* (I5)

(c) Initial challenges for sharing on the social network

With the start of social media posts, mothers also faced obstacles. Publicly exposing their child’s frailties would mean sharing moments full of adaptations and vulnerabilities with people. Due to the population’s lack of knowledge about toxoplasmosis, such exposure could mean subjecting their families to questioning, rejection, and the opinions of others. The daily commitments and intensive care routine required by the diagnosis, especially at the beginning of their journey on the social network, made sharing even more challenging. Faced with such difficulties, some mothers chose to temporarily withdraw from social media, using this offline moment as an essential part of their journey of understanding after receiving the diagnosis: *“But I didn’t touch it for a while, because I was recovering, right? My mental health, I was doing therapy, so I didn’t post anything for a while, then I came back.”* (P11)

#### 3.2.2. Analytical Theme 2: Social Networking as an Ally for Achieving Multiple Goals

The empowerment found on social networks to overcome and cope with their child’s diagnosis encouraged mothers to share their own experiences. Thus, social media provided continuity in interactively coping with toxoplasmosis, going through challenges, fears, and stigmas, and becoming a mission.

(a) Social media posts: missions and themes shared

The participants reported that the initial aim of the posts was to help the public understand the reality of toxoplasmosis, with posts that addressed the mother’s experience throughout the diagnosis process. The mothers also often shared their difficulties in dealing with the emotions of receiving the diagnosis, mainly because of the comparisons they made with idealizations of typical motherhood. At this point, posts aimed at combating prejudice gained even more strength. In these posts, the mothers emphasized that toxoplasmosis could become a reality for anyone when not prevented, regardless of social class or living conditions:

*“But I intended to help others, to help other mothers who needed some guidance, and it worked really well… I felt I had a mission to help other mothers.”* (P6)

*“But in the eyes of society, I became the atypical mother. This can happen to anyone, at any time, and make your child’s path lighter.”* (I11)

(b) Rays of hope: sharing family life and new possibilities

Over time, social media posts focused on treatment, conveying hope, and sharing experiences to help other mothers. These posts aimed to demystify negative views about toxoplasmosis, showing that it is possible to have a “typical” family routine and ensuring that the child, even with limitations, is treated with dignity. By highlighting similarities and differences in the cases of diagnosis, the mothers found new ways to strengthen the bonds with their children, emphasizing that atypical motherhood is full of love and happy moments despite the challenges:

*“I think it shows that the routine, despite atypical motherhood, is a typical routine. So, in the same way, my husband and I can have our routine of work, study, and leisure with two daughters, one who will always need more care, but it’s a normal routine in which you can carry on with your life.”* (P2)

*“But looking at these photos and videos from October, I can see that we’re managing to insert outings, parties, and games into this rollercoaster we live on. [She] has the right to play too, and my fight is to provide the best for her.”* (I9)

(c) Valuing reality through the exchange of experiences

Sharing routines and progress related to toxoplasmosis allowed new mothers on this journey to find a point of reference to compare their child’s development. This sharing of experiences made the burden lighter and facilitated the identification of possible treatments and therapies. A support network was thus formed between mothers from different social backgrounds, driven by the will to overcome challenges and share knowledge, transcending the barriers of inequality. This constant interaction on social media has led mothers to adopt a new relationship with these platforms. However, this can sometimes be challenging due to the need to manage time and daily tasks and it not being a paid activity: *“Is it tiring for me? Yes, I don’t earn a dollar from it, I don’t earn, I don’t earn, and I won’t earn. It’s not about earning money; I earn from my work, you know? It’s to bring information, to bring love, you know?”* (P4)

#### 3.2.3. Analytical Theme 3: A Reason to Keep Going: Turning Goals into Actions

The recognition and identification made possible by posting on social networks created a network of support and encouragement, strengthening the motivation to maintain accounts and posts related to the diagnosis.

(a) Attracting different followers

The increase in shares made it possible to expand the connections beyond family and friends, reaching other people interested in the subject, especially other mothers and families of children with the same diagnosis. This expansion occurred mainly due to the use of hashtags related to toxoplasmosis, which made it easier for other mothers to find information, who also became part of the group of followers who followed and were inspired by these mothers’ routines. The growing reach of the material posted on these profiles allowed the network to expand beyond Brazil, crossing geographical boundaries in the digital context, overcoming physical barriers, and enabling the mothers’ mission to be even more widespread: *“Many parents have already called me because I put the hashtag toxoplasmosis or low vision, precisely because I needed to. So, today I try to put it so other parents can come.”* (P3)

(b) United by purpose: followers’ interests

The followers began actively following the posts, using them as support to face their difficulties. During the routines portrayed, the mothers noticed increased public demonstrations of love and care for their children. In this context, these mothers became a point of reference for many followers. Furthermore, other mothers discovering the diagnosis in their pregnancy used the networks for guidance, protection, and support. Interest in planning, organizing, and optimizing care routines was also present, especially concerning the use of devices and tools to support daily care that enhances the child’s abilities and functional capacities: *“Well, I’m following the guidelines of the professionals who accompany me and understand the subject—pediatrics and speech therapy. So, we’re starting the introduction of feeding with the feeding pacifier… all very calmly.”* (I9)

(c) “It takes a village”: creating a community through social networks

The sum of the elements shared by the mothers and the followers’ interests transformed social media into its most profound meaning by creating a network of support and social support between mothers, helping to reframe their journeys beyond the screens. This expansion of social networks beyond the virtual also made it possible for mothers to receive more significant support from their own families, who could be present, even if only virtually, to celebrate their achievements. The reach of the networks and sharing opinions and experiences with new followers also contributed to positive experiences related to motherhood amid the chaos. In this way, they strove to share their experiences more effectively, inspiring their followers:

*“He’s had reels where people tag [other people]: ‘See, you can do it, there’s a way, there’s evolution.’ So that’s very good for me. Because that was missing [for me], you know?”* (P4).

*“I think the internet and social networks are very effective mechanisms for people to get to know each other. It’s a huge exchange: ‘I went to such and such a doctor. I solved it with this medicine.’”* (P9)

(d) Lessons learned from their social media journeys

When they realize they have succeeded in helping people, going beyond the limits imposed by their busy routines and the fear of prejudice, the mothers feel a sense of mission accomplishment. From this experience, they believe they have found an assertive and fruitful path. The encouragement created by the posts also helps strengthen the support network they have built up and their persistence in caring for their children, working as a system that feeds on experiences. Looking back on their journeys, mothers often realize how many personal challenges they have overcome regarding caring for their children and managing their networks. They then continue their journey through optimistic efforts in the face of the transformations brought about by the diagnosis: *“Because there are a lot of mothers who give up. I have mothers who don’t do therapy with their children because they’ve given up the ghost, because they don’t think it’s going to work. ‘Oh, but are you going to spend the rest of your life doing it?’ Yes, it’s for the rest of your life, but if you don’t do it, it’s not going to work.”* (P2)

To sum up, mothers facing the diagnosis of congenital toxoplasmosis in their children found refuge in social networks to deal with the initial lack of knowledge and shock. They sought information and support, created profiles dedicated to the disease, and even faced challenges, such as public exposure to vulnerabilities and the intensive care routine. Their posts sought to raise awareness of toxoplasmosis and highlight the importance of a “normal” life despite limitations, inspiring other mothers and raising awareness of their child’s daily achievements. This sharing of experiences generated a community of support, both virtual and real, expanding beyond geographical borders. Followers found the mothers’ posts a source of motivation and reference in their journeys. Moreover, the mothers saw their actions’ positive impact, strengthening and encouraging them to continue. Thus, social networks became a space for sharing transformation and mutual support, giving new meaning to the journeys of mothers and their families.

## 4. Discussion

The objective of this study was to analyze the motivations of mothers of children with congenital toxoplasmosis to create Instagram^®^ accounts and share their experiences and daily lives, investigating what meaning they attribute to creating these profiles and how they relate to caring for their diagnosed children. This research allowed us to find different relationships between the psychosocial, emotional, and informational motivations that drive this online behavior, shaped mainly by the broad audience that their posts have reached.

Motherhood is a journey full of challenges, and when it comes to atypical children, the challenges can be even more significant [11]. Receiving a diagnosis that classifies a child as atypical is an overwhelming moment for mothers, impacting not only the way they see their child’s future but also their own identity and psychological well-being. This process of assimilation can be accompanied by a range of emotions, permeated by uncertainty and helplessness, leading to the need to understand more about the diagnosis [23,24]. Thus, one of the motivations for sharing life in the virtual world was the need to find information about the disease, coupled with the limited availability of reliable information about the diagnosis of congenital toxoplasmosis. This unexpected context, which is challenging to cope with, can also lead to changes in various aspects of family life, which can include the denial of the diagnosis [25,26]. In addition, there is a breakdown of expectations of a healthy child in the family, with the possibility of changes in daily life, permeated by the use of medication, frequent tests, and treatments, as well as physical, psychological, and social limitations [27]. Thus, as reported by the mothers in the study and evidenced by the evaluation of the accounts, the publications created on Instagram^®^ allowed for the sharing of different perspectives on the disease. The possibility of seeing photos and honest accounts of other mothers going through similar situations helped them adapt to the new reality.

The mothers found on their Instagram^®^ accounts a space to connect with others facing similar situations. By sharing their stories, especially in their first posts, they sought solidarity and empathy from an online community that understood the challenges of being diagnosed with congenital toxoplasmosis. Experiences of online communities of mothers revealed that emotional support is offered through a sense of belonging, building bonds, expressing feelings, sharing messages of support, praise, and encouragement, and providing interactions between mothers [15]. It also stood out as a space for sharing information to empower women [15].

In some cases, families also experience financial changes, as the treatment of chronic illnesses can be expensive, including medical expenses, therapies, medication, and even hospitalizations. It is worth mentioning that, due to their full-time dedication to caring for the child, many caregivers have to quit their jobs or, to access essential health services, they have to travel or seek treatments that require more significant financial resources [28]. As shown in this study, creating a community through Instagram^®^ also allowed mothers to connect with families from different socioeconomic backgrounds, promoting social aid to those in need. Their posts allowed them to share their experiences and disseminate knowledge that, for many, would be inaccessible without their presence on social media.

With the emergence and worsening of the child’s health situation, which consequently demands more dependence and full-time dedication from the caregiver [29], the often lonely and exhausting family routine, physically and mentally, interferes with the quality of life of mothers, and a lack of support causes overload and can lead to emotional and social impacts [30]. Effective support networks are necessary to preserve the quality of life of caregivers of people facing disabilities and chronic illnesses, and they also serve as sources of information and guidance related to the diagnosis [30]. In addition, the support given by friends and family, and the manifestations of faith and spirituality, were very present throughout the publications, indicated by the mothers as driving forces that guide their journey on the social network. This presence helps them to face and overcome the challenges caused by the disease and can make the process lighter and more positive [26].

The process of being diagnosed with the disease involves moments of acceptance and readjustment of various aspects of family life, including psychosocial aspects [26]. This support, found on Instagram^®^, has awakened a sense of purpose in helping other mothers and families in this process of discovering the disease. Mothers see the role of the caregiver as defending the rights of children, and do their best to provide quality care [31,32]. It is a challenging process, filled with uncertainties, discoveries, changes, and daily struggles. Many give up their professional and social lives to dedicate themselves fully to caring for their child. Relationships become limited to a few family members, health professionals, and other people who identify with the moment they are facing, which is the main profile of followers on social media. When they find welcoming relationships, mainly from other people experiencing similar situations, caregivers re-signify their roles with feelings of usefulness and importance, sharing experiences, emotions, and difficulties. This sharing brings comfort, satisfaction, support, enthusiasm, and belonging. Thus, mothers or caregivers understand that this network promotes self-esteem and adds value to themselves, creating bonds and mutual support [31,32]. In this study, sharing provided them with a sense of accomplishment, and they felt that they were doing something meaningful by creating a place that offered support and information, also contributing to their emotional resilience. In addition, by posting about congenital toxoplasmosis to their followers, they felt empowered to advocate for their child’s rights and needs and influence other women and mothers. Their empowerment strengthened the family, sharing the child’s achievements and progress on this journey, showing that the routine can be normal, even if it presents specific needs.

Mothers also saw online sharing as an opportunity to educate the general public about the risks of the disease and ways to prevent it, as well as the process faced from diagnosis to treatment, and the challenges faced by their families. Children with chronic illnesses can face stigmatization and discrimination at school, in the community, and in social settings, which can limit their full participation and inclusion in society, and the fear of this exposure can prevent a peaceful experience, as children are subject to aversive consequences, such as insults and contempt. Furthermore, a lack of awareness and understanding of chronic illnesses in children can result in a lack of empathy and adequate support for families [33]. The online publications also aimed to break the taboo, reduce the stigma, and promote a more comprehensive understanding of this condition.

Conducting the interviews alongside the virtual ethnographic analysis of social networks represented an essential methodological tool to strengthen the findings and delve deeper into the meanings and motivations for sharing experiences on social media. However, we acknowledge some limitations of the study, such as the exclusive focus on Instagram, which may only capture some of the mothers’ experiences on social media. Focusing exclusively on Portuguese-language accounts may limit insights into mothers’ experiences in other linguistic or cultural contexts. Additionally, cultural nuances may affect how experiences and motivations are expressed and perceived. Furthermore, the mothers who chose to participate in the research were mainly from Brazil’s South and Southeast regions and were more engaged and proactive on social media, which may not reflect the experiences of those less active online or with limited internet access.

Despite these limitations, the study has significant implications for practice and research. Future research could expand the sample to include different regions, cultures, and languages, including exploring the role of religious practices, allowing for a more comprehensive view of mothers’ experiences. Additionally, comparative studies between other social media platforms could provide insights into how communication and support vary among them. Research should also explore how findings can be translated into practical strategies for healthcare professionals. Understanding how social media influences maternal support and knowledge can help develop targeted interventions and support systems, informing public policies and health programs aimed at caring for children with congenital infections by supporting their families. It can also raise awareness among healthcare professionals about the context and challenges these families face, helping to identify their needs. The creation and involvement of healthcare professionals in social media may also benefit mothers, guaranteeing their access to reliable information and a reference for consultation.

## 5. Conclusions

We found that mothers and families of children with congenital toxoplasmosis formed a community on Instagram^®^, where they shared their successful experiences, the positive aspects of motherhood, and the challenges they face daily, finding emotional support, sharing information, and developing support networks. They also created meaningful friendships and contributed to greater awareness and acceptance of diversity concerning children’s special needs in their communities and society in general. In addition to sharing the characteristics of the disease and teaching and sensitizing the population about ways to prevent infection during pregnancy, their posts promoted a broader reach to people beyond families facing the same situation and led the public to view the condition of children with congenital toxoplasmosis with empathy, resilience, and support.

## Data Availability

The original contributions presented in the study are included in the article, further inquiries can be directed to the corresponding author.
